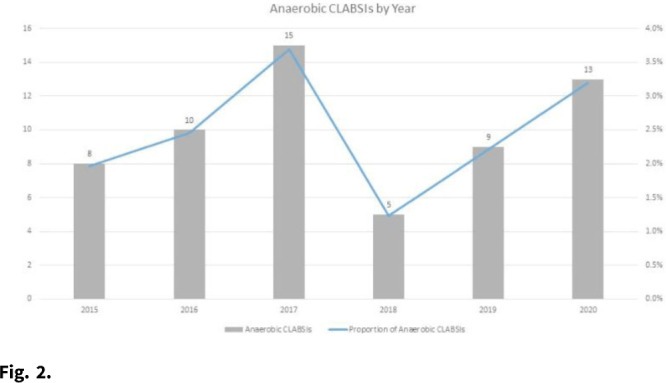# Central-line associated bloodstream infections secondary to strict anaerobes: Time for A definition change?

**DOI:** 10.1017/ash.2022.197

**Published:** 2022-05-16

**Authors:** Jessica Seidelman, Sarah Lewis, Ibukun Kalu, Erin Gettler, Sonali Advani, Deverick Anderson, Becky Smith

## Abstract

**Background:** Central-line–associated bloodstream infections (CLABSIs) arise from bacteria migrating from the skin along the catheter, by direct inoculation, or from pathogens that form biofilms on the interior surface of the catheter. However, given the oxygen-poor environments that obligate anaerobes require, these organisms are unlikely to survive long enough on the skin or on the catheter after direct inoculation to be the true cause of a CLABSI. Although some anaerobic CLABSIs may meet the definition for a mucosal-barrier-injury, laboratory-confirmed, bloodstream infection (MBI-LCBI), some may be not. We sought to determine the proportion of CLABSIs attributed to obligate anaerobic bacteria, and we sought to determine the pathophysiologic source of these infections. **Methods:** We performed a retrospective analysis of prospectively collected CLABSI data at 54 hospitals (academic and community) in the southeastern United States from January 2015 to December 2020. We performed chart reviews on a convenient sample for which medical records were available. We calculated the proportion of CLABSIs due to obligate anaerobes, and we have described a subset of anaerobic CLABSI cases. **Results:** We identified 60 anaerobic CLABSIs of 2,430 CLABSIs (2.5%). Of the 60 anaerobic CLABSIs, 7 were polymicrobial with nonanaerobic bacteria. The most common species we identified were *Bacteroides*, *Clostridium*, and *Lactobacillus* (Table 1). The proportion of anaerobic CLABSIs per year varied from 1.2% to 3.7% (Fig. 1). Of 60 anaerobic CLABSIs, 29 (48%) occurred in the only quaternary-care academic medical center in the database. In contrast, an average of 0.6 (SD, 0.6) anaerobic CLABSIs occurred in the 53 community hospitals over the 6-year study period. Of these 29 anaerobic CLABSIs, 23 (79%) were clinically consistent with secondary bloodstream infections (BSIs) due to gastrointestinal or genitourinary source, but they lacked appropriate documentation to meet NHSN criteria for secondary BSI or MBI-LCBI based on case reviews by infection prevention physicians. The other 6 anaerobic CLABSIs did not have a clear clinical etiology and did not meet MBI-LCBI criteria. In addition, 27 (93%) of 29 anaerobic CLABSIs occurred in patients who were either solid-organ transplant recipients, were stem-cell transplant recipients, or were receiving chemotherapy. Lastly, 27 (93%) of 29 anaerobic CLABSIs were treated with antibiotics. **Conclusions:** Anaerobic CLABSIs are uncommon events, but CLABSI may disproportionately affect large, academic hospitals caring for a high proportion of medically complex patients. Additional criteria could be added to the MBI-LCBI to better classify anaerobic BSI.

**Funding:** None

**Disclosures:** None

**Figure:**
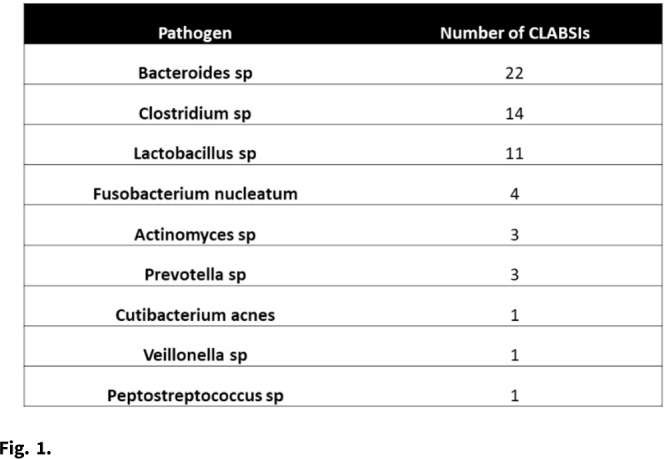

**Figure:**